# Efficacy of robot-assisted fingers training in chronic stroke survivors: a pilot randomized-controlled trial

**DOI:** 10.1186/s12984-015-0033-5

**Published:** 2015-04-25

**Authors:** Evan A Susanto, Raymond KY Tong, Corinna Ockenfeld, Newmen SK Ho

**Affiliations:** Interdisciplinary Division of Biomedical Engineering, The Hong Kong Polytechnic University, Hong Kong, S.A.R. China; Department of Electronic Engineering Division of Biomedical Engineering, The Chinese University of Hong Kong, Hong Kong, S.A.R. China

**Keywords:** Stroke, Stroke recovery, Rehabilitation, Fingers, Chronic stroke, Robotic rehabilitation

## Abstract

**Background:**

While constraint-induced movement therapy (CIMT) is one of the most promising techniques for upper limb rehabilitation after stroke, it requires high residual function to start with. Robotic device, on the other hand, can provide intention-driven assistance and is proven capable to complement conventional therapy. However, with many robotic devices focus on more proximal joints like shoulder and elbow, recovery of hand and fingers functions have become a challenge. Here we propose the use of robotic device to assist hand and fingers functions training and we aim to evaluate the potential efficacy of intention-driven robot-assisted fingers training.

**Methods:**

Participants (6 to 24 months post-stroke) were randomly assigned into two groups: robot-assisted (robot) and non-assisted (control) fingers training groups. Each participant underwent 20-session training. Action Research Arm Test (ARAT) was used as the primary outcome measure, while, Wolf Motor Function Test (WMFT) score, its functional tasks (WMFT-FT) sub-score, Fugl-Meyer Assessment (FMA), its shoulder and elbow (FMA-SE) sub-score, and finger individuation index (FII) served as secondary outcome measures.

**Results:**

Nineteen patients completed the 20-session training (Trial Registration: HKClinicalTrials.com HKCTR-1554); eighteen of them came back for a 6-month follow-up. Significant improvements (p < 0.05) were found in the clinical scores for both robot and control group after training. However, only robot group maintained the significant difference in the ARAT and FMA-SE six months after the training. The WMFT-FT score and time post-training improvements of robot group were significantly better than those of the control group.

**Conclusions:**

This study showed the potential efficacy of robot-assisted fingers training for hand and fingers rehabilitation and its feasibility to facilitate early rehabilitation for a wider population of stroke survivors; and hence, can be used to complement CIMT.

## Background

Stroke remains the leading cause of severe long-term disabilities worldwide, with hemiplegia associated with abnormal muscle activation and coordination, muscle weaknesses, spasticity, and loss of dexterity and precision being the major contributors to the disabilities [1-7]. While almost 70% of stroke survivors are able to regain walking ability within the first six months, recovery of the paretic upper-extremity is still challenging [8-11]. Approximately only 38% of stroke patients regained some dexterity in their paretic arm six months post-stroke [12,13]. Even more demanding is the recovery of hand and fingers functions, which is proven very limited with no consistent pattern of improvement [14,15].

A recent review has suggested the use of constraint-induced movement therapy (CIMT) and robot-assisted therapy for hand rehabilitation [14,15]. CIMT is a rehabilitation therapy during which the participant was asked to complete different tasks with his/her non-paretic limb being constrained; hence forcing the use of the paretic limb itself. By doing so, it is expected that the learned non-use process can be prevented [16-18]. Currently, CIMT is arguably one of the most promising methods for upper limb rehabilitation post-stroke with studies showing impressive improvements after training [15,16,18-21]. However, there have been debates that the high efficacy shown in CIMT studies is mainly attributed to the very selected population of stroke survivors who are less impaired and/or able to tolerate prolonged constraint [14]. The very intense and strenuous nature of CIMT seems to limit its applicability to general stroke survivors.

Robotic devices, on the other hand, have been adept complements to conventional therapy due to their ability to facilitate repetitive movement training with high intensity and precision [14,15] as well as their applicability to wider audience of the stroke population. Many of them can be customized to meet patient’s needs, making them more suitable for stroke survivors with wide range of impairment level.

Researchers have developed different robotic rehabilitation devices to reduce the neurological impairment of the upper limb after stroke; IntelliArm, MIT MANUS, and ARMin are just three examples of the growing collections of rehabilitation robots with various features [22-24]. Nevertheless, many of these devices still focus on functional rehabilitation of the more proximal joints, such as shoulder and elbow. In 2007, we developed a hand exoskeleton robot system that facilitates movements of each individual finger in both flexion and extension directions [25-28]. A recent review indicates this as one of the only few devices with such feature [29]. Similar result was observed by Heo et al. in their review on hand exoskeleton technologies [30].

This study explores the possibility of implementing the robot-assisted rehabilitation for finger dexterity recovery after stroke and evaluates the effects of its instructed assistance control algorithm. We investigate the possible efficacy of this robot-assisted fingers training over a 20-session rehabilitation program and the long-term effect of this training on the paretic upper limb over a six-month follow-up.

## Methods

### Device and control algorithm

The hand exoskeleton robot used in this study was originally designed by our group [27], with some modifications to suit the purpose of the study by enabling active individual finger control via joint moment sensing [28]. This device, having 5 individual digits powered by 5 linear actuators (Firgelli L12, Firgelli Technologies, Inc.), allows simultaneous flexion of 55 and 65 degrees around the metacarpophalangeal (MCP) and proximal interphalangeal (PIP) joints, respectively. The device was attached to user’s paretic hand using Velcro straps.

**Figure 1:**
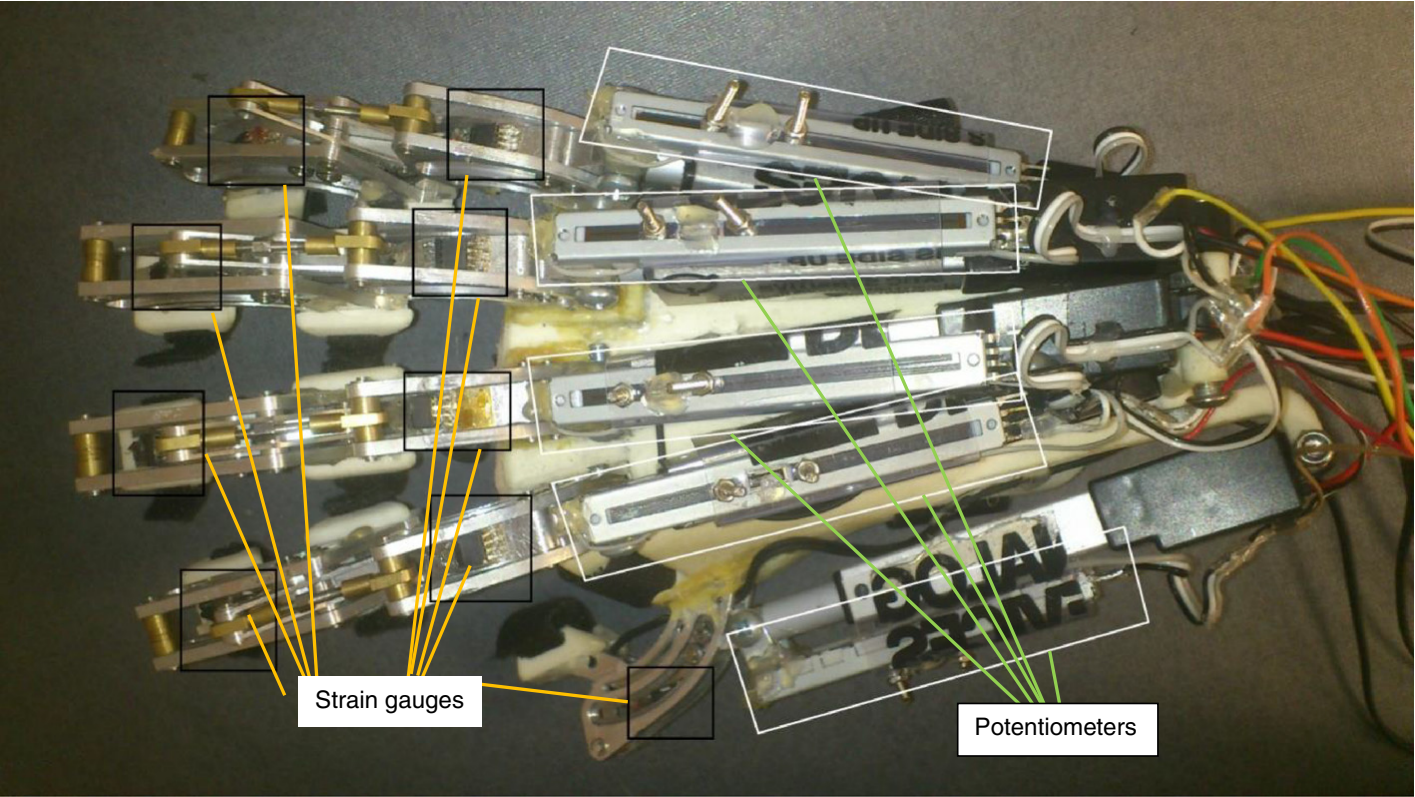
The modified hand exoskeleton robot. This is a picture of the hand exoskeleton robot after modification. Highlighted in black squares are the strain gauges mounted to the device for joint moments measurement, while highlighted in white squares are the linear potentiometers.

Nine full-bridge strain-gauges ZF1000-2 EB-T (Shenzhen Nanhua Electronic Technology Co., Ltd., China) were mounted to measure MCP and PIP joint moments of every finger; five sliding potentiometers RS6011Y1401A (Alps Electric Co., Ltd., USA) were installed to provide each finger’s position feedback (see Figure 1) [28].

The joint moment signals were amplified 1000 times and, together with the position feedback signals, were then inputted into NI-USB 6218 DAQ (National Instruments, Corp., USA) to be sampled at 1000 Hz sampling rate and 16-bit resolution. All data were processed using LabVIEW (National Instruments, Corp., USA).

The device provided assistance to the user’s paretic hand to accomplish three gestures: hand grasping and opening, three-finger (thumb, index, and middle finger) pinching and opening, and two-finger (thumb and index finger) pinching and opening. For the pinching gestures, non-instructed fingers were kept in flexed position.

The control algorithm was designed such that, the device would initiate its assistance when the MCP joint moments of all the instructed fingers were above the threshold level, and those of the non-instructed fingers were below the threshold level. With average enslaving force, which is the force produced by a non-instructed finger while another finger is exerting maximum force, in individuals with stroke being around 25% of its respective maximum force [31], we set the threshold for each finger at 20% of its own MCP joint flexion and extension isometric maximum voluntary torque (MVT) measured prior to each training session.

The direction of the assistance (i.e. flexion or extension) was pre-determined and instructed to the subjects; the system automatically reversed the direction after the digit reached the target position, as indicated by the potentiometers.

Depending on the purpose of the use, the linear actuators of the device are detachable from the exoskeleton digits, allowing those digits to move freely with only minimum friction from the mechanical parts. This would be useful for: (1) performing non-assistive hand training, and (2) measuring the user’s active range-of-motion (ROM).

### Subjects

Stroke survivors are eligible to participate if they satisfy the following: (1) primary stroke 6 to 24 months prior to the beginning of the intervention, (2) moderate stroke condition (50 > FMA score > 20) [32,33], (3) ability to understand simple commands (Mini Mental State Examination score > 21), and (4) ability to differentiate sensation on one finger from the other fingers. They are excluded if they have: (1) recurrent stroke, (2) other neurological, neuromuscular, orthopedic disease, or (3) shoulder or arm contracture/pain.

### Randomization

Participants were randomized into 2 groups: (1) the robot-assisted (robot) group and (2) the non-assisted (control) group, with 1:1 ratio, by random number generator.

### Protocol

Participants underwent a total of 20 one-hour sessions of robot-assisted fingers training. The training intensity was set at 3 to 5 times a week with all 20 sessions to be completed within 5 consecutive weeks. The human subjects ethics review for this study had been approved by the Departmental Research Committee of the Hong Kong Polytechnic University.

In every session, the participant was seated comfortably and stretching on his/her upper-limb was done passively by a physical therapist for 10 minutes. The hand exoskeleton robot was then put on the subject’s paretic hand with his elbow positioned at 90° flexion and his forearm on an arm-rest. The subject was subsequently asked to perform maximum flexion and extension of each digit individually for 3 seconds in a randomized order to measure their MVTs.

The training was performed without the arm-rest, and comprised three modes: hand grasp, three-finger pinch, and two-finger pinch. In all three modes, the subjects were instructed to move a kitchen sponge on a horizontal plane of the table in front of them. Four points were marked on the table, in the shape of a rhombus with horizontal and vertical diagonals of 500 mm and 300 mm. The movement started from the paretic side, to the non-paretic side, forward, backward, then back to the paretic side. This movement was performed for about 4 minutes with full hand grasp, 8 minutes with three-finger pinch, and another 8 minutes with two-finger pinch. A short break (1–2 minutes) was allowed after each part of the training. The robot group completed this section with the device’s assistance. The control group, on the other hand, completed the exact same task within the same time frame without any assistance from the device as the linear actuators of the instructed fingers were disconnected from the exoskeleton digits as described earlier. Assistance from the therapist, however, was provided for the control group whenever deemed necessary throughout the training session.

Continuous verbal instructions and postural control by the therapist were used to minimize compensation by the non-paretic arm throughout the session.

### Outcome measures

Action Research Arm Test (ARAT) was adopted as the primary efficacy outcome measure because of its high reliability (r > 0.9) and due to its ability to assess not only proximal control of the arm, but also its dexterity [34-36]. This assessment has an overall maximum score of 57 and can be divided into 4 subsets: grasp, grip, pinch, and gross movement. However, it was suggested that these subset scores should never be used independently due to the uni-dimensional nature of the test [37].

As the secondary outcome measures, Wolf Motor Function Test (WMFT) and Fugl-Meyer Assessment (FMA), which are as reliable as the ARAT (r > 0.9) [33-36,38-41], as well as session-to-session finger independency index (FII) were used. WMFT was intended to quantify UE functional ability in stroke patients based on the performance and the time required to complete joint motions and functional tasks [38]. The WMFT consists of: 15 tasks (6 joint segment tasks, 9 functional tasks; maximum score = 75), each of which should be performed within 120 seconds, and 2 strength measurements [38,41]. As the training focused on dexterity, the sub-score of WMFT consisting the 9 functional tasks only (WMFT-FT) was also analyzed as a separate measure to evaluate the subjects’ improvement in terms of functional tasks ability. FMA was designed as performance-based impairment index to assess the motor function, balance, sensations, and joint functions in hemiplegic stroke survivors [33]. In this study, FMA refers only to the upper extremity motor function part with a total score of 66. Additionally, in order to specifically evaluate functional changes in the proximal and distal joints, the shoulder and elbow subset score of the FMA (FMA-SE; maximum score = 36), and the wrist and hand subset score of the FMA (FMA-WH; intraclass correlation coefficient = 0.97 [42]; maximum score = 24) were also analyzed separately.

Four measurements of WMFT, ARAT, and FMA were taken: within 2 weeks from the first training session (Pre1), within 1 week from the first session(Pre2), within 3 days after the last session (Post), and 6-months follow-up (6Mo). The study was single-blinded so the assessors were of no knowledge of the grouping.

Meanwhile, FII is measured on every 5 sessions and defined as follows:

$$ FII = {\displaystyle \sum_{i=1}^4}\frac{F{I}_i}{4} $$

$$ F{I}_i = \frac{\overset{\hbox{---} \hbox{---} }{F_{l,l}}}{{\displaystyle {\sum}_{j=1}^4}\overset{\hbox{---} \hbox{---} }{F_{i,j}}} $$

$\overset{\hbox{---} \hbox{---} }{F_{i,j}}$ indicates the maximum flexion force of finger *j* while finger *i* is doing an isometric flexion MVT and $\overset{\hbox{---} \hbox{---} }{F_{l,l}}$ indicates the maximum flexion force of finger *i* during MVT. *i* and *j* indicate the index of the four fingers other than thumb, from the index finger (1) to the little finger (4).

Both FI and FII range from 0 to 1. An FI value of 0 indicates a complete inability of the instructed finger to exert flexion force, while FI value of 1 indicates the ability of the instructed finger to perform flexion MVT with the other 3 fingers exerting no flexion force at all. As the average value of the four fingers’ FIs, FII represents, in general, the individuality of the four fingers.

### Data analysis

Statistical analysis of all the outcome measures data was conducted using the non-parametric tests. Wilcoxon’s Signed-Rank tests were done to evaluate functional changes within each group at different time points, and Mann–Whitney U-test was performed to compare the two groups in terms of functional improvement right after the intervention (Pre2-Post) and 6 months after the intervention (Pre2-6Mo), with Pre2 data as the baseline. Change in outcome measures is considered significant if the p-value is less than 0.05. Intention-to-treat principle was used.

Mean change of FMA and ARAT scores were compared against their estimated minimal clinically important difference (MCID) values; and WMFT mean change against its estimated minimal detectable change (MDC) value [36,39,40]. Proportions of participants exceeding these MCID/MDC values were also calculated.

## Results

**Figure 2:**
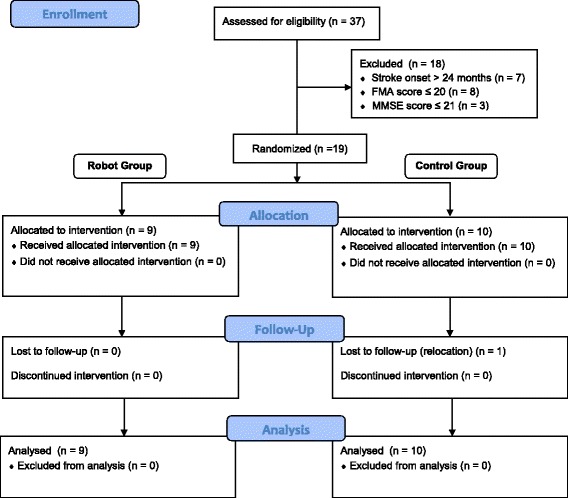
CONSORT patient flow throughout the study.

The clinical trial was registered to HKClinicalTrials (http://www.hkclinicaltrials.com) with a unique identifier of HKCTR-1554. From January until September 2013, 37 stroke survivors responded to our recruitment; eighteen of them did not meet our recruitment criteria (see Figure 2 for more details). The nineteen stroke survivors (14 males and 5 females, aged 53.2 ± 9.9 years old) who met the requirements were recruited, gave informed consent, and were randomly distributed into 2 groups (9 to the robot group and 10 to the control group). All of them completed the intervention, and only one from the control group did not take the follow-up assessment due to relocation. The demographic data of the participants is shown in Table 1. No significant bias was found between the two groups with respect to age, gender, handedness, affected side, stroke type, and mean months from the onset to the first training session.

**Table 1:** **Patients’ demographic data**

**Characteristic**	**Robot (n = 9)**	**Control (n = 10)**
**Mean age [SD]**	50.7 [9.0]	55.1 [10.6]
**Sex, male (%)**	7(78)	7(70)
**Handedness, right (%)**	9(100)	10(100)
**Affected side, right (%)**	3(33)	4(40)
**Stroke type, hemorrhagic (%)**	3(33)	5(50)
**Mean months from onset to first training session [SD]**	16.4 [5.8]	16.1 [5.1]

Summary of the statistical analysis is shown in Table 2. Baseline values were quite stable with no significant difference found between Pre-1 and Pre-2. After intervention, significant improvements of ARAT, WMFT score, WMFT time, WMFT-FT score, WMFT-FT time, and FMA-SE were present in the robot group; and significant improvements of ARAT, WMFT score, FMA, FMA-SE and FMA-WH were found in the control group. However, only the robot group was able to maintain significant differences in ARAT and FMA-SE scores, six months after training.

**Table 2:** **Intra-group comparisons of clinical assessment scores**

**Outcome Measures**	**Mean ± SD**				**p-value**	
	**Pre-1**	**Pre-2**	**Post**	**6-Mo**	**Pre-2 vs. Post**	**Pre-2 vs. 6-Mo**
**Robot**						
**ARAT**	16.56 ± 10.86	17.33 ± 10.62	31.33 ± 8.01	28.33 ± 11.97	0.008*	0.044*
**WMFT Score**	34.56 ± 8.37	35.33 ± 8.54	44.89 ± 10.77	42.56 ± 9.03	0.007*	0.109
**WMFT Time**	53.78 ± 18.00	51.44 ± 20.67	36.54 ± 18.61	34.04 ± 15.76	0.011*	0.066
**WMFT-FT Score**	10.22 ± 6.27	11.22 ± 7.44	20.11 ± 7.99	17.67 ± 7.89	0.007*	0.123
**WMFT-FT Time**	89.51 ± 24.46	86.16 ± 31.14	55.78 ± 27.47	56.58 ± 28.23	0.008*	0.066
**FMA**	31.67 ± 12.19	31.89 ± 11.98	37.00 ± 12.48	38.00 ± 13.53	0.065	0.123
**FMA-SE**	18.44 ± 7.40	17.89 ± 7.43	21.33 ± 6.82	21.56 ± 7.95	0.012*	0.020*
**FMA-WH**	10.56 ± 5.12	11.11 ± 5.30	12.56 ± 4.52	13.78 ± 5.16	0.438	0.210
**Control**						
**ARAT**	18.60 ± 9.88	20.80 ± 8.30	28.50 ± 5.95	27.40 ± 8.78	0.014*	0.083
**WMFT Score**	35.10 ± 5.43	35.40 ± 4.00	40.40 ± 6.50	38.30 ± 6.86	0.027*	0.107
**WMFT Time**	49.60 ± 15.83	47.15 ± 18.42	43.52 ± 12.55	44.47 ± 13.91	0.333	0.445
**WMFT-FT Score**	12.70 ± 4.00	14.40 ± 3.47	16.80 ± 4.77	15.60 ± 5.28	0.085	0.550
**WMFT-FT Time**	76.54 ± 29.21	71.44 ± 26.90	67.22 ± 20.58	70.00 ± 26.53	0.333	0.959
**FMA**	33.30 ± 6.78	34.60 ± 8.16	40.30 ± 7.54	37.30 ± 9.72	0.008*	0.083
**FMA-SE**	20.50 ± 4.22	20.50 ± 5.37	23.80 ± 5.33	21.90 ± 6.02	0.012*	0.230
**FMA-WH**	10.30 ± 3.20	11.30 ± 3.29	13.30 ± 2.49	12.10 ± 3.70	0.018*	0.255

Abbreviations: ARAT, Action Research Arm Test; WMFT, Wolf Motor Function Test; WMFT-FT, the functional movement tasks of Wolf Motor Function Test; FMA, Fugl-Meyer Assessment; FMA-SE, the shoulder and elbow parts of FMA; FMA-WH, the wrist and hand parts of FMA.

*indicates significant difference.

Inter-group comparisons showed significantly better WMFT-FT score and time improvements post-training (both with p = 0.017) in the robot group (see Table 3). No significant difference between the two groups was present in any of the clinical scores 6 months after training.

**Table 3:** **Inter-group comparisons of post-intervention effects**

**Outcome Measures**	**Mean Change ± SD**		**p-value**
**Improvement**	**Robot**	**Control**	
**Pre2-Post**			
**ARAT**	14.00 ± 5.75	7.70 ± 6.91	0.053
**WMFT Score**	9.56 ± 7.54	5.00 ± 6.46	0.113
**WMFT Time**	−14.91 ± 12.06	−3.63 ± 10.96	0.079
**WMFT-FT Score**	8.89 ± 8.67	2.40 ± 4.12	0.017*
**WMFT-FT Time**	−30.38 ± 23.74	−4.22 ± 21.01	0.017*
**FMA**	5.11 ± 6.55	5.70 ± 4.35	0.968
**FMA-SE**	3.44 ± 2.01	3.30 ± 2.65	0.905
**FMA-WH**	1.44 ± 4.14	2.00 ± 1.67	0.484
**Pre2-6Mo**			
**ARAT**	11.00 ± 13.91	6.60 ± 11.09	0.497
**WMFT Score**	7.22 ± 12.50	2.90 ± 5.07	0.720
**WMFT Time**	−17.40 ± 24.10	−2.68 ± 8.80	0.156
**WMFT-FT Score**	6.44 ± 11.26	1.20 ± 3.71	0.356
**WMFT-FT Time**	−29.58 ± 39.92	−1.44 ± 12.42	0.095
**FMA**	6.11 ± 10.90	2.70 ± 4.42	0.604
**FMA-SE**	3.67 ± 5.35	1.40 ± 2.87	0.356
**FMA-WH**	2.67 ± 4.97	0.80 ± 1.99	0.565

Abbreviations: ARAT, Action Research Arm Test; WMFT, Wolf Motor Function Test; WMFT-FT, the functional movement tasks of Wolf Motor Function Test; FMA, Fugl-Meyer Assessment; FMA-SE, the shoulder and elbow parts of FMA; FMA-WH, the wrist and hand parts of FMA.

*indicates significant difference.

As shown in Table 4, the average change in WMFT, ARAT, and FMA scores of the robot group were higher than the MCID/MDC values both post-training and at 6-month follow-up, while only ARAT is higher than MCID/MDC in the control group. Similarly, proportions of stroke survivors exceeding the MCID/MDC values were higher in the robot group in all clinical scores.

**Table 4:** **Comparison of intervention effects against MCID/MDC**

**Outcome Measures**	**MCID/MDC**	**Pre2-Post**		**Pre2-6Mo**	
		**Robot**	**Control**	**Robot**	**Control**
**Mean Change**					
**ARAT**	5.70	14.00*	7.70*	11.00*	6.60*
**WMFT Score**	5.55	9.56*	5.00	7.22*	2.90
**WMFT Time**	−4.36	−14.91*	−3.63	−17.40*	−2.68
**FMA**	4.25	5.11*	5.70*	6.11*	2.70
**Proportion Exceeding MCID/MDC**					
**ARAT**	5.70	9/9(100%)	6/10(60%)	6/9(67%)	5/10(50%)
**WMFT Score**	5.55	8/9(89%)	4/10(40%)	4/9(44%)	2/10(20%)
**WMFT Time**	−4.36	8/9(89%)	6/10(60%)	6/9(67%)	5/10(50%)
**FMA**	4.25	5/9(56%)	5/10(50%)	5/9(56%)	4/10(40%)

Abbreviations: ARAT, Action Research Arm Test; WMFT, Wolf Motor Function Test; FMA, Fugl-Meyer Assessment; MCID, Minimal Clinically Important Difference; MDC, Minimal Detectable Change.

*indicates average improvement higher than MCID/MDC.

**Figure 3:**
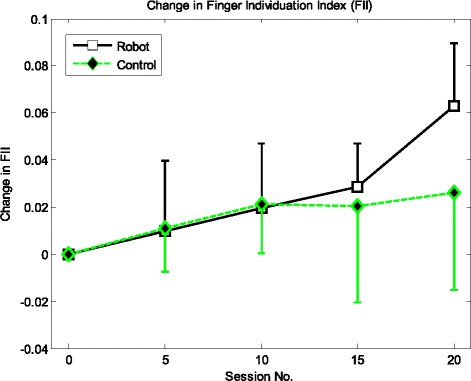
The change in FII throughout the 20-session training. The black solid line indicates the more obvious FII improvement of the robot group as compared to that of the control group indicated by the green dashed line.

Figure 3 shows an increasing trend of FII improvement in the robot group all the way to the last session, while FII improvement in the control group seemed to be reaching a plateau after the 10th session. In spite of such difference, non-parametric test showed no significant difference between FII in the first session and that in the last session in both groups (p = 0.096 for the robot group and p = 0.527 for the control group), nor did it show significant difference on FII improvement in the inter-group comparison (p = 0.400).

Additionally, in terms of repetitions made (accumulated from all three modes: grasping, three-finger pinching, and two-finger pinching) by the subjects during the specified training period, the robot group significantly increased their total repetitions from 80.56 ± 23.23 repetitions during the first training session to 109.11 ± 9.41 repetitions in the last session (p = 0.004). Similarly, the control group also gained significant increase from 62.13 ± 17.96 repetitions initially to 83.63 ± 22.12 repetitions by the last training session (p = 0.002). The robot group, however, performed significantly more repetitions by the last training session (p = 0.006) despite the insignificant difference between the two groups in the first session (p = 0.090).

## Discussion

Applying robot-assisted therapy to fingers dexterity rehabilitation, the results showed better improvement of fingers dexterity in the robot group when compared to the control. Significant improvements in the robot group were observed in most of the outcome measures post training and 44% to 67% of the participants in that group experienced improvements exceeding the respective MCID/MDC of each clinical score after 6 months. Compared to the control group, which represents conventional therapy with comparable intensity but without any assistance from the device, the robot group has a significantly better recovery after 20 sessions of training.

Nevertheless, we attributed the better improvements of the robot group to two factors. First, the encouraged use of the hand and selected fingers in the training early in the chronic stroke phase (6 to 24 months after stroke onset) seems to promote reversal of the behaviorally reinforced learned non-use due to repetitive movements of the affected hand and fingers, just like how this mechanism happened to the paretic upper limb in general after the forced use of it in CIMT studies [19,21]. We believe this is also accompanied by expansion of the paretic arm representation area in the primary motor cortex, despite direct evidence unavailable. This factor, also shared by the control group, also explains the slight improvement in the control group. This is supported by the fact that in the last training sessions, the subjects in both groups performed significantly more repetitions of the tasks than they did during the first session.

Second is the control algorithm, which is unique to the robot group and appears to be the difference maker. The control algorithm that requires instructed fingers to be active and uninstructed fingers to relax might have provided the robot group with feedbacks that promote motor learning and muscle coordination. The control group, who is not provided with such feedbacks, does not seem to share similar benefit.

The significant differences found in FMA-SE in both groups may suggest improvements on shoulder and elbow joints as well despite the device mainly focused on hand and fingers. The involvement of other joints in the tasks might be beneficial for the whole upper limb, albeit at different levels. With proximal to distal gradient of motor deficit being absent [43], this result further suggests that holistic approach of rehabilitation, as opposed to joint per joint rehabilitation, is the way to go. This suggestion is in line with the findings of Oujamaa et al., who suggests that exercising the more distal joints of the paretic arm is essential and efficient [44].

Furthermore, due to robot assistance, we anticipated a possible intensity decrease, and thus reduced efficacy in the robot group, compared to the non-assisted control group. This case, however, was not evident; on the contrary, the incorporation of the device seemed to give positive effect to the training as subjects in robot group may have compensated the reduced intensity due to the robot assistance with more repetitions and less use of non-paretic arm support throughout the training; resulting in increased actual use of their paretic arm and subsequently promoting improvement-induced increased use outside the sessions. The results showed significantly higher number of repetitions performed in the last session with robotic training.

While significant improvements were found in ARAT and FMA-SE in the robot group at the 6-month follow-up time point, the inter-group difference, however, was insignificant. This is most likely due to the large variations among the subjects. Larger sample size may be necessary to show the significant difference after 6 months.

Moving forward, we are of the belief that robot-assisted rehabilitation should be applied in combination with CIMT. While CIMT would be very beneficial for those who can handle it, it requires relatively high baseline to start with; effectively limiting its target group. This robot-assisted fingers training, on the other hand, appears to be beneficial for a wider group of patients with moderate to severe impairment level after stroke when compared to CIMT. Hence, a rehabilitation protocol started as early as possible with robot-assisted training and later followed by CIMT, we believe, would better benefit individuals with stroke.

Having mentioned that, we also consider the need of establishing a clinical score boundary, above which individuals with stroke shall be recommended to enroll in CIMT and below which robot-assisted intervention shall be given to them, in the future studies. In this study, we have shown that the robot-assisted training were able to facilitate stroke survivors with minimum WMFT score of 24. Meanwhile for CIMT, with the pooled WMFT prescore means of 44.85, and considering the stringent nature of CIMT, we estimate a bottom line of around 35 to 40 is necessary for a stroke survivor to be able to undergo CIMT. Once this boundary is established, a sequential robot-assisted therapy and CIMT, like the one studied by Hsieh et al. [45], can be systematically applied in clinical settings.

Limitations of this study also deserve to be addressed. The main limitation of this study is that this is just a pilot study with limited sample size; nevertheless, this study should suffice to estimate the necessary sample size for a full-scale study. Secondly, as the non-paretic arm of the participants is not being constrained, its use might vary across subjects and result in a high variability of the improvement. Lastly, with a literature which reviewing 66 studies on upper limb functional rehabilitation after stroke [44], authors were recommending 30 hours functional rehabilitation training for upper limb for chronic stroke rehabilitation. The intensity applied in this study might have room for further improving the efficacy of the training, since the finger individuation index (FII) showed continuous improvement between the 15 and 20 training sessions. More training sessions/hours should be considered in future studies.

## Conclusions

While full-scale study is still needed for confirmation, this study has shown the potential of robot-assisted fingers training to enhance upper limb function in general, and hand and fingers functions in particular. Our findings also suggest that upper-limb rehabilitation shall be done holistically by using tasks that involve multiple joints as one functional unit instead of focusing on just one or two joints only.

Additionally, to prevent loss of hand and expansion of non-hand representation areas in the primary motor cortex and to prevent learned non-use from setting in, CIMT, and rehabilitation training in general, must be started as early as possible. With CIMT requiring high baseline, this robot-assisted therapy, which can facilitate earlier rehabilitation for individual with stroke, proved to be a viable option to bridge the gap and be a good complement for CIMT.

## References

[1] Mackay J, Mensah GA (2004). The Atlas of Heart Disease and Stroke.

[2] WHO (2003). The World Health Report 2003: Shaping The Future.

[3] Dobkin BH (2004). Strategies for stroke rehabilitation. Lancet Neurol.

[4] Lang CE, Wagner JM, Bastian AJ, Hu Q, Edwards DF, Sahrmann SA, Dromerick AW (2005). Deficits in grasp versus reach during acute hemiparesis. Exp Brain Res.

[5] Nowak D, Grefkes C, Dafotakis M, Küst J, Karbe H, Fink GR (2007). Dexterity is impaired at both hands following unilateral subcortical middle cerebral artery stroke. Eur J Neurosci.

[6] Hermsdörfer J, Hagl E, Nowak D, Marquardt C (2003). Grip force control during object manipulation in cerebral stroke. Clin Neurophysiol.

[7] Cirstea MC, Mitnitski AB, Feldman AG, Levin MF (2003). Interjoint coordination dynamics during reaching in stroke. Exp Brain Res.

[8] Kwakkel G, Wagenaar RC, Twisk JW, Lankhorst GJ, Koetsier JC (1999). Intensity of leg and arm training after primary middle-cerebral-artery stroke: a randomised trial. Lancet.

[9] Nakayama H, Jørgensen HS, Raaschou HO, Olsen TS (1994). Recovery of upper extremity function in stroke patients: the Copenhagen Stroke Study. Arch Phys Med Rehabil.

[10] Go AS, Mozaffarian D, Roger VL, Benjamin EJ, Berry JD, Blaha MJ (2014). Heart disease and stroke statistics--2014 update: a report from the American heart association. Circulation.

[11] Kelly-Hayes M, Beiser A, Kase CS, Scaramucci A, D’Agostino RB, Wolf PA (2003). The influence of gender and age on disability following ischemic stroke: the Framingham study. J Stroke Cerebrovasc Dis.

[12] Kwakkel G, Kollen BJ, van der Grond J, Prevo AJH (2003). Probability of regaining dexterity in the flaccid upper limb: impact of severity of paresis and time since onset in acute stroke. Stroke.

[13] Kwakkel G, Kollen BJ, Krebs HI (2008). Effects of robot-assisted therapy on upper limb recovery after stroke: a systematic review. Neurorehabil Neural Repair.

[14] Langhorne P, Coupar F, Pollock A (2009). Motor recovery after stroke: a systematic review. Lancet Neurol.

[15] Langhorne P, Bernhardt J, Kwakkel G (2011). Stroke rehabilitation. Lancet.

[16] Taub E (1976). Movement in nonhuman primates deprived of somatosensory feedback. Exerc Sport Sci Rev.

[17] Taub E, Crago JE, Burgio LD, Groomes TE, Cook EW, DeLuca SC, Miller NE (1994). An operant approach to rehabilitation medicine: overcoming learned nonuse by shaping. J Exp Anal Behav.

[18] Taub E, Crago JE, Uswatte G (1998). Constraint-induced movement therapy: a new approach to treatment in physical rehabilitation. Rehabil Psychol.

[19] Taub E, Uswatte G, Elbert T (2002). New treatments in neurorehabilitation founded on basic research. Nat Rev Neurosci.

[20] Wolf SL, Thompson P, Winstein CJ, Miller JP, Blanton SR, Nichols-Larsen DS, Morris DM, Uswatte G, Taub E, Light KE, Sawaki L (2010). The EXCITE Stroke Trial. Comparing Early and Delayed Constraint-Induced Movement Therapy. Stroke.

[21] McIntyre A, Viana R, Janzen S, Mehta S, Pereira S, Teasell R (2012). Systematic review and meta-analysis of constraint-induced movement therapy in the hemiparetic upper extremity more than six months post stroke. Top Stroke Rehabil.

[22] Ren Y, Kang SH, Park H-S, Wu Y-N, Zhang L-Q (2013). Developing a multi-joint upper limb exoskeleton robot for diagnosis, therapy, and outcome evaluation in neurorehabilitation. In IEEE Trans neural Syst Rehabil Eng.

[23] Krebs HI, Volpe BT, Williams D, Celestino J, Charles SK, Lynch D, Hogan N (2007). Robot-aided neurorehabilitation: a robot for wrist rehabilitation. In IEEE Trans Neural Syst Rehabil Eng.

[24] Nef T, Quinter G, Müller R, Riener R (2009). Effects of arm training with the robotic device ARMin I in chronic stroke: three single cases. Neurodegener Dis.

[25] Tong KY, Ho SK, Pang PK, Hu XL, Tam WK, Fung KL, Wei XJ, Chen PN, Chen M (2010). An intention driven hand functions task training robotic system. Conf Proc IEEE Eng Med Biol Soc.

[26] Ho NSK, Tong KY, Hu XL, Fung KL, Wei XJ, Rong W (2011). An EMG-driven exoskeleton hand robotic training device on chronic stroke subjects: task training system for stroke rehabilitation. IEEE Int Conf Rehabil Robot.

[27] Hu XL, Tong KY, Wei XJ, Rong W, Susanto EA, Ho SK (2013). The effects of post-stroke upper-limb training with an electromyography (EMG)-driven hand robot. J Electromyogr Kinesiol.

[28] Susanto EA, Tong RKY, Ho NSK, Hu XL (2012). Hand Exoskeleton Robot as a Force Measurement Tool. 9th IASTED Int Conf Biomed Eng.

[29] Maciejasz P, Eschweiler J, Gerlach-Hahn K, Jansen-Troy A, Leonhardt S (2014). A survey on robotic devices for upper limb rehabilitation. J Neuroeng Rehabil.

[30] Heo P, Gu GM, Lee SJ, Rhee K, Kim J (2012). Current hand exoskeleton technologies for rehabilitation and assistive engineering. Int J Precis Eng Manuf.

[31] Li S, Latash ML, Yue GH, Siemionow V, Sahgal V (2003). The effects of stroke and age on finger interaction in multi-finger force production tasks. Clin Neurophysiol.

[32] Velozo C, Woodbury ML (2011). Translating measurement findings into rehabilitation practice: an example using Fugl-Meyer Assessment-Upper Extremity with patients following stroke. J Rehabil Res Dev.

[33] Fugl-Meyer AR, Jääskö L, Leyman I, Olsson S, Steglind S (1975). The post-stroke hemiplegic patient. 1.a method for evaluation of physical performance. Scand J Rehabil Med.

[34] Platz T, Pinkowski C, van Wijck F, Kim I-H, di Bella P, Johnson G (2005). Reliability and validity of arm function assessment with standardized guidelines for the Fugl-Meyer Test, Action Research Arm Test and Box and Block Test: a multicentre study. Clin Rehabil.

[35] Hsueh IP, Lee MM, Hsieh CL (2002). The Action Research Arm Test: is it necessary for patients being tested to sit at a standardized table?. Clin Rehabil.

[36] Van der Lee JH, De Groot V, Beckerman H, Wagenaar RC, Lankhorst GJ, Bouter LM (2001). The intra- and interrater reliability of the action research arm test: a practical test of upper extremity function in patients with stroke. Arch Phys Med Rehabil.

[37] Koh CL, Hsueh IP, Wang WC, Sheu CF, Yu TY, Wang CH, Hsieh CL (2006). Validation of the action research arm test using item response theory in patients after stroke. J Rehabil Med.

[38] Wolf SL, Catlin PA, Ellis M, Archer AL, Morgan B, Piacentino A (2001). Assessing wolf motor function test as outcome measure for research in patients after stroke. Stroke.

[39] Page SJ, Fulk GD, Boyne P (2012). Clinically important differences for the upper-extremity Fugl-Meyer Scale in people with minimal to moderate impairment due to chronic stroke. Phys Ther.

[40] Lin KC, Hsieh YW, Wu CY, Chen CL, Jang Y, Liu JS (2009). Minimal detectable change and clinically important difference of the wolf motor function test in stroke patients. Neurorehabil Neural Repair.

[41] Morris DM, Uswatte G, Crago JE, Cook EW, Taub E (2001). The reliability of the wolf motor function test for assessing upper extremity function after stroke. Arch Phys Med Rehabil.

[42] Page SJ, Levine P, Hade E (2012). Psychometric properties and administration of the wrist/hand subscales of the Fugl-Meyer Assessment in minimally impaired upper extremity hemiparesis in stroke. Arch Phys Med Rehabil.

[43] Beebe JA, Lang CE (2008). Absence of a proximal to distal gradient of motor deficits in the upper extremity early after stroke. Clin Neurophysiol.

[44] Oujamaa L, Relave I, Froger J, Mottet D, Pelissier J-Y (2009). Rehabilitation of arm function after stroke. Literature review. Ann Phys Rehabil Med.

[45] Hsieh Y-W, Lin K-C, Horng Y-S, Wu C-Y, Wu T-C, Ku F-L (2014). Sequential combination of robot-assisted therapy and constraint-induced therapy in stroke rehabilitation: a randomized controlled trial. J Neurol.

